# Comprehensive analysis of published phase I/II clinical trials between 1990-2010 in osteosarcoma and Ewing sarcoma confirms limited outcomes and need for translational investment

**DOI:** 10.1186/2045-3329-2-5

**Published:** 2012-01-27

**Authors:** Annemiek M van Maldegem, Aparna Bhosale, Hans J Gelderblom, Pancras CW Hogendoorn, Andrew B Hassan

**Affiliations:** 1 Department of Oncology, Oxford Cancer and Haematology Centre, Churchill Hospital, University of Oxford, Oxford OX3 7LJ, UK; 2 Sir William Dunn School of Pathology, South Parks Road, University of Oxford, Oxford OX1 3RE, UK; 3 Department of Clinical Oncology, Leiden University Medical Center, Leiden, PO Box 9600, 2600 RC Leiden, The Netherlands; 4 Department of Pathology, Leiden University Medical Center, Leiden, PO Box 9600, 2600 RC Leiden, The Netherlands

**Keywords:** Ewing sarcoma, osteosarcoma, early phase trials, translational research

## Abstract

**Background:**

High grade primary bone sarcomas are rare cancers that affect mostly children and young adults. Osteosarcoma and Ewing sarcoma are the most common histological subtypes in this age group, with current multimodality treatment strategies achieving 55-70% overall survival. As there remains an urgent need to develop new therapeutic interventions, we have reviewed published phase I/II trials that have been reported for osteosarcoma and Ewing sarcoma in the last twenty years.

**Results:**

We conducted a literature search for clinical trials between 1990 and 2010, either for trials enrolling bone sarcoma patients as part of a general sarcoma indication or trials specifically in osteosarcoma and Ewing sarcoma. We identified 42 clinical trials that fulfilled our search criteria for general sarcoma that enrolled these patient groups, and eight and twenty specific trials for Ewing and osteosarcoma patients, respectively. For the phase I trials which enrolled different tumour types our results were incomplete, because the sarcoma patients were not mentioned in the PubMed abstract. A total of 3,736 sarcoma patients were included in these trials over this period, 1,114 for osteosarcoma and 1,263 for Ewing sarcoma. As a proportion of the worldwide disease burden over this period, these numbers reflect a very small percentage of the potential patient recruitment, approximately 0.6% for Ewing sarcoma and 0.2% for osteosarcoma. However, these data show an increase in recent activity overall and suggest there is still much room for improvement in the current trial development structures.

**Conclusion:**

Lack of resources and commercial investment will inevitably limit opportunity to develop sufficiently rapid improvements in clinical outcomes. International collaboration exists in many well founded co-operative groups for phase III trials, but progress may be more effective if there were also more investment of molecular and translational research into disease focused phase I/II clinical trials. Examples of new models for early translational and early phase trial collaboration include the European based EuroBoNeT network, the Sarcoma Alliance for Research through Collaboration network (SARC) and the new European collaborative translational trial network, EuroSarc.

## Background

Primary bone sarcomas are rare, and account for approximately 6% of all childhood malignancies, with Ewing sarcoma and osteosarcoma accounting for approximately 3% of tumours arising in teenagers. Here we focus on osteosarcoma and Ewing sarcoma, the two most common bone sarcomas, whose treatments are similar as they involve multimodality treatment with dose intensive and toxic chemotherapy, combined with potentially mutilating surgery.

Osteosarcoma is the most common primary malignant tumour arising from bone. It is a pleiomorphic tumour of bone, and based on animal model systems, thought to arise in mesenchymal stem cells in which the mutant proliferating spindle cells produce osteoid or immature bones [1]. The EMEA published data in 2009 estimating approximately five people in 1,000,000 in the European Union (EU) are affected by osteosarcoma. Recently, data from the RARECARE surveillance network revealed an incidence of 0.23 per 100,000, amounting to approximately 1135 new cases per year in EU27 [2]. Osteosarcoma mostly affects children and young adults with the median age of diagnosis being fifteen. Seventy-five percent of patients are between eight and twenty-five years old. Osteosarcoma is often located in the extremities of long bones near metaphyseal growth plates. Current treatments for osteosarcoma achieve 60-70% event-free survival (EFS) for patients who present with localized disease and approximately 20% EFS for patients with clinically detectable metastatic disease [3]. Of all patients, 20% have clinically detectable metastatic disease at first presentation. Surgical resection of all clinically detectable sites of disease and systemic therapy to control microscopic metastatic disease is currently the therapy of choice for early stage osteosarcoma. Since the introduction of neo-adjuvant and adjuvant chemotherapy to surgery in the early 1980s, the long term survival of patients with osteosarcoma has remained stable at about 60-65%. In terms of chemotherapy, several agents have demonstrated activity in osteosarcoma including cisplatin, doxorubicin, high-dose methotrexate with leucovorin rescue (HDMTX) and ifosfamide. For current treatment options these agents are combined. Since the early 1980s, trials have been conducted in which the variations in doses and scheduling between these four agents were tested, but these have not result in improvement of EFS [4]. Since the introduction of ifosfamide, more than two decades ago, the only new agent that has been approved is muramyl tripeptide, a drug that activates the innate immune system [5]. Thus, despite surgical resection of the primary tumour and aggressive adjuvant chemotherapy, 30%-40% still die of metastases that are resistant to conventional therapies [6-8]. For osteosarcoma patients with resectable pulmonary metastases it has become more standard to treat these patients with metastectomy. This has been shown to improve relapse free survival and a subgroup of patients may even be cured [9].

Ewing Sarcoma/Primitive Neuroectodermal (PNET) are the second most common bone malignancy after osteosarcoma in children and young adults with a peak incidence at age fifteen. Ewing sarcoma are diagnostically defined by a Ewing sarcoma EWS (Chromosome 22) translocation resulting in fusion with an ETS transcription factor, the commonest abnormality (85%) being EWS-FLI1 (Chromosome 11) or rarely with a non ETS family partner [10,11]. Although claimed in the past that the transcript type reflected a difference in prognoses [12] this proved not to be the case in prospective randomised trials [13]. RARECARE estimates an EU27 incidence rate for bone and soft tissue Ewing sarcoma of 0.13 per 100,000 and 0.05 per 100,000, respectively [2]. This translates to approximately 647 bone and 263 soft tissue new Ewing sarcoma diagnoses per year (EU27), with a predicted 65-75% 5 year survival for non-metastatic disease using conventional chemotherapy, radiotherapy, high dose chemotherapy and peripheral blood stem cell transplant (PBSCT). However, survival for the 25% of patients that present with metastatic disease is approximately 20% [14], and for those who develop relapsed and/or refractory disease, the survival is no more that 10%. To date, studies in the patient population requiring salvage treatment have been confined to chemotherapy combinations and high dose chemotherapy, with variable response rates and little or no impact on survival. During the last two decades, the outcome has improved in patients with localized disease. This has been achieved by dose intensification and standardisation of conventional therapeutics and radiotherapy. With the use of multidisciplinary treatments, such as chemotherapy (including vincristine, doxorubicin, cyclophosphamide, ifosfamide and etoposide), surgery and radiation therapy, the five year overall survival probability exceeds 75% for patients with non metastatic low volume Ewing sarcoma. However, advances in the treatment of Ewing sarcoma have not impacted on the outcome of patients with large volume and metastatic disease [15].

In the last two decades the treatment outcome for these bone sarcomas has not improved greatly, even though some new treatment interventions have been successfully tested. Large scale phase III trials with long durations of recruitment have established material for prognostic and treatment related correlative studies with survival and toxicity outcomes, and importantly, have formed the basis of international collaboration. However, the smaller phase I/II studies are also important in order to develop proof of principle single agent and combination treatments, particularly with newer molecular and biological based interventions that directly test disease specific molecular mechanisms.

We wished to establish the effective level of phase I/II activity that has been reported in peer reviewed publications in the last twenty years. We report an overview of the phase I/II trials that have been conducted for osteosarcoma and Ewing sarcoma, where we detail the kind of drugs that have been tested, what the published study outcomes were and what interventions have progressed to testing in phase III trials. The results show an improvement in overall activity, but that the number of studies and International collaborations in early phase trials remain at a low and limited level. We discuss potential routes to improve the number and quality of early phase trials in Ewing and osteosarcoma.

## Methods

### Search strategy

We report data available in the public domain only. Publications were identified from searches of PubMed, the Cochrane Controlled Trials Register, American Society of Clinical Oncology (ASCO) abstracts and ClinicalTrials.gov. databases for the period 1990-2010. The search strategies used terms for osteosarcoma; (osteosarcoma) AND (phase I OR phase II) AND (clinical trial), and was supplemented with a text word search. For Ewing sarcoma the search algorithm was; (Ewing sarcoma) AND (phase I OR phase II) AND (clinical trial). To validate the search we broadened the search algorithm to; (sarcoma) AND (phase I OR phase II) AND (clinical trial), and compared the results from the narrow search with the ones from the broad search. The latest search was performed in April 2010.

Whenever multiple reports from the same trial were published, we used only the report with the longest follow-up to avoid any duplication of information. Publications were eligible if they: (1) described (or cited a paper that described) osteosarcoma or Ewing sarcoma study of early phase clinical trials; (2) were published in English; and (3) came from industrialized countries. All types of evaluation were accepted (full papers, conference abstracts, reports) as long as results (including data) were presented.

### Data extraction

Data extraction was conducted independently by two authors (A. B. and A.M. van M.). We used a systematic method for the search normally used for meta-analysis [16]. Differences in data extraction were resolved by consensus with a third author (A.B. H.). From each eligible trial we recorded authors' names, journal and year of publication, number of patients enrolled, number of osteosarcoma or Ewing sarcoma patients, study phase and the outcome of the trial.

## Results and discussion

### Eligible trials

**Figure 1:**
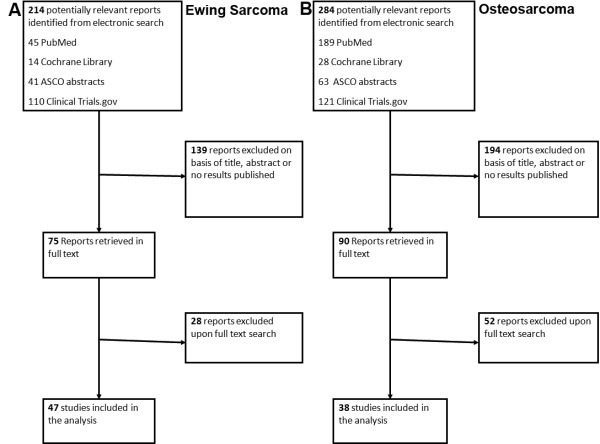
**Flowchart diagrams of the clinical trial selection criteria outcomes for Ewing sarcoma (**A**) and osteosarcoma (**B**)**.

A flow-chart indicating the identification of clinical trials for inclusion in the analysis is reported for Ewing sarcoma (Figure 1A) and osteosarcoma (Figure 1B). During the search many reports had to be excluded because no results were published. When we searched the reports using full text, we had to exclude some papers because neither osteosarcoma nor Ewing sarcoma patients were included in these studies. When we combined the results, we identified 42 trials enrolling patients with any histological diagnosis of sarcoma that were eligible for our study, in that they included osteosarcoma and Ewing sarcoma. Of the 42 clinical trials twenty-one were phase I, two were phase I/II and nineteen were phase II trials. We found eight clinical trials which included only Ewing sarcoma patients; of this group two were phase I and six were phase II trials. We identified twenty trials that included only osteosarcoma patients. There were two phase I, sixteen were phase II and two were phase I/II trials. A total of 3,736 patients were included in all the clinical trials, of which 1,263 were Ewing sarcoma and 1,114 were osteosarcoma patients.

### Primary Outcome

**Figure 2:**
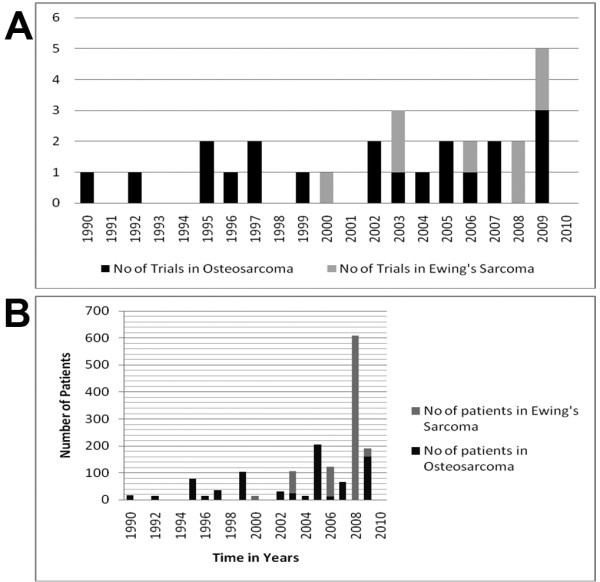
**Distribution of the number of trials published that have recruited either osteosarcoma or Ewing sarcoma patients between 1990-2010 (**A**)**. The total number of either osteosarcoma or Ewing Sarcoma patients entered in phase I/II trials published between 1990-2010 (**B**).

Tables 1 and 2 show the search results for clinical trials conducted in patients with the wider diagnosis of general sarcoma, and specifically the trials that included either osteosarcoma (Table 3) or Ewing sarcoma patients only (Table 4). Table 1 and 2 are trials testing either chemotherapy or biological treatments, respectively. Analysis of the number of trials conducted which included only osteosarcoma or Ewing sarcoma patients from 1990 to the present, (Figure 2A) it was clear that the number of trials reported for osteosarcoma has been stable since 1995, with approximately five trials in five years. For Ewing sarcoma there has been an increase in the number of trials published, with the no early phase trials reported between 1990 and 1999, an increasing number of trials in the period 2000-2005 and even more between 2006-2010.

**Table 1:** Characteristics of eligible trials for general sarcoma with chemotherapy based treatment for phase I and phase II

Author	Year	Intervention	No Osteo patients	No Ewing Patients	Phase	Outcome
**Pratt et al**, Cancer, 74: 2593-8 [22]	1994	Leucovorin, 5-Fluorouracil	22	12	II	Ewing: 3 (25%) SD, Osteo: 1 (5%) SD

**Kushner et al**, J Clin Oncol, 13 (11): 2796-804 [23]	1995	Cyclo, Doxo, Etop, Ifos, Vin	Not mentioned	Not mentioned	II	6 (17%) CR, 2 (6%) PR, 34 (95%) CIR

**Antman et al**, Cancer, 82 (7): 1288-95 [24]	1998	Doxo, Dacarbazine, Ifos	31	13	II	10 (12%) CR, 34 (42%) PR

**Blaney et al**, Clin Cancer Res, 4 (2): 357-60 [25]	1998	Topotecan	18	25	II	1 (1%) CR, 1 (1%) PR, 8 (9%) SD

**Lucidarme et al**, Bone Marrow Transplant, 22 (6): 535-40 [26]	1998	Thiotepa combined with SCR	7	3	II	11 (50%) PR

**Berg et al**, J Pediatric Hematol Oncol, 22 (6): 506-9 [27]	2000	Pyrazoloacridine	8	10	II	0% response

**Delaloge et al**, J Clin Oncol, 19 (5): 1248-55 [28]	2001	Trabectedin	3	1	I	4 (14%) CR, 10 (35%) SD

**Saylors et al**, J Clin Oncol, 19 (15): 3463-9 [29]	2001	Cyclo and Topotecan	18	17	II	6 (35%) OR

**Wagner et al**, Clin Cancer Res, 10 (3): 840-8 [30]	2004	Temozolomide, Irinotecan	0	7	I	1 (8%) CR, 2 (17%) PR

**Lau et al**, Clin Cancer Res, 11: 672-7 [31]	2005	Trabectedin	4	3	I	1 (8%) CR

**van Winkle et al**, Pediatr Blood Cancer, 44 (4): 338-47 [32]	2005	Ifos, Carboplatin and Etop	35	22	I/II	Not mentioned

**Hawkins et al**, Pediatr Blood Cancer, 47 (6): 328-37 [33]	2006	Topotecan	11	20	II	2 (4%) PR

**Navid et al**, Cancer, 106 (8): 1846-56 [34]	2006	Vin, Doxo, Cyclo, Ifos and Etop	0	11	II	47 (66%) CR

**Wagner-Bohn et al**, Cancer, 46 (2): 262 [35]	2006	Gemcitabine	2	4	II	2 (10%) SD

**Zaucha et al**, Int J Rad Oncol Biol Phys, 64 (1): 227-34 [36]	2006	Total body radiation after high-dose chemotherapy	0	10	II	13 (25%) CR, 10 (19%) PR

**Zwerdling et al**, Cancer, 106 (8): 1821-8 [37]	2006	Docetaxel	23	21	II	2 (1%) CR, 6 (3%) PR, 17 (10%) SD

**Geoerger et al**, J Clin Oncol, 26: 4394-400 [38]	2008	Oxaliplatin	6	2	I	2 (4%) PR, 17 (6%) SD

**Geler et al**, Pediatr Blood Cancer, 52	2009	Paclitaxel, Ifos	1	3	I	3 (20%) PD, 5 (33%) SD

**Wagner et al**, ASCO abstract	2009	Vin, Irinotecan, Temozolomide	3	5	I	1 (3%) PR,2 (6%) OR

**McGregor et al**, Cancer, 115 (8): 1765-75 [39]	2009	Oxaliplatin, Irinotecan	6	1	I	1 (8%) CR, 1 (8%) SD

Abbreviations: ClR = Clinical Response; CR = Complete Response; Cyclo = cyclophosphamide; Doxo = doxorubicine; EFS = Event Free Survival; Etop = etoposide; Ewing = Ewing Sarcoma; Ifos = ifosfamide; No = number; OR = Objective Response; Osteo = Osteosarcoma; PD = Progressive Disease; PR = Partial Response; RR = Response Rate; SCR = Stem Cell Rescue; SD = Stabile Disease; TMR = Total Marrow Radiation; Vin = vincristine

**Table 2:** Characteristics of eligible trials for general sarcoma with biologically based treatment for phase I and phase II

Author	Year	Intervention	No Osteo Patients	No Ewing Patients	Phase	Outcome
**Daw et al**, J Clin Oncol, 23 (25): 6172-80 [40]	2005	Gefitinib	6	3	I	1 (4%) PR, 4 (16%) SD

**Maki et al**, Cancer, 103 (7): 1431-8 [41]	2005	Bortezomib	1	2	II	1 (4%) OR

**Villablanca et al**, J Clin Oncol, 24 (21): 3423-30 [42]	2006	Fenretinide	2	5	I	1 (2%) CR, 13 (24%) SD

**Biron et al**, ASCO abstract	2007	Gimatecan	0	Not mentioned	II	3 (8%) SD

**Bagatell et al**, Clin Cancer Res, 13 (6): 1783-8 [43]	2007	Tanespimycin	6	2	I	0% OR

**Jimeno et al**, Pediatr Blood Cancer, 49 (3): 352-7 [44]	2007	Gefitinib	3	1	I	1 (7%) PR, 3 (20%) SD

**Kramer et al**, J Clin Oncol, 25 (34): 5465-70 [45]	2007	Monoclonal antibody(131-I-3F8)	0	2	I	3 (23%) ClR

**Bond et al**, Pediatr Blood Cancer, 50 (2): 254-8 [46]	2008	Imatinib mesylate	10	24	II	1 (1%) PR

**Chao et al**, ASCO abstract	2008	Imatinib mesylate	0	7	II	1 (14%) PR

**Fox et al**, Clin Cancer Res, 14 (4): 1111-5 [47]	2008	Tubulin Inhibitor (ABT-751)	3	3	I	0% OR

**Jakacki et al**, J Clin Oncol, 26 (30): 4921-7 [48]	2008	Erlotinib, Temozolomide	1	0	I	1 (2%) SD

**Langevin et al**, Pediatr Blood Cancer, 50 (3): 577-80 [49]	2008	Rebeccamycin	16	14	II	% OR

**Mita et al**, J Clin Oncol, 26 (3): 361-7 [50]	2008	Deforolimus	1	1	I	4 (13%) PR, 18 (56%) SD

**Olmos et al**, ASCO abstract	2008	Figitumumab	0	9	I	1 (5%) PR, 6 (27%) SD

**Chawla et al**, Mol Ther, 17 (9): 1651-7 [51]	2009	Rexin-G	3	1	I/II	13 (65%) SD

**Chugh et al**, J Clin Oncol, 27 (19): 3148-53 [52]	2009	Imatinib	27	13	II	1 (0.5%) CR, 3 (2%) PR

**Malempati et al**, ASCO abstract	2009	Cixutumumab	Not mentioned	10	I	1 (4%) PR

**Patel et al**, ASCO abstract	2009	IGF-1R antibody (R1507)	43	71	II	Not mentioned

**Widemann et al**, J Clin Oncol, 27 (4): 550-6 [53]	2009	Ixabepilone	3	2	I	4 (21%) SD

**Jacobs et al**, Clin Cancer Res, 16 (2): 750-4 [54]	2010	Ixabepilone	11	9	II	0% OR

**Kurzrock et al**, Clin Cancer Res, 16 (8): 2458-65 [55]	2010	IGF-1R antibody (R1507)	0	9	I	2 (5%) PR, 13 (35%) SD

**Olmos et al**, Lancet Oncol, 11: 129-35 [56]	2010	Figitumumab	0	16	I	1 (3%) CR, 1 (3%) PR, 8 (28%) SD

*Abbreviations*:, ClR = Clinical Response; CR = Complete Response; Cyclo = cyclophosphamide; Doxo = doxorubicin; EFS = Event Free Survival; Etop = etoposide; Ewing = Ewing Sarcoma; Ifos = ifosfamide; No = number; OR = Objective Response; Osteo = Osteosarcoma; PR = Partial Response; RR = Response Rate; SCR = Stem Cell Rescue; SD = Stabile Disease; TMR = Total Marrow Radiation; Vin = vincristine

**Table 3:** Characteristics of eligible trials for osteosarcoma only

Author	Year	Intervention	Phase	No of Patients	Outcome
**Salesh RA., et al., Cancer **65:861-5.	1990	Etoposide 72-h i.v 600 mg/m^2^. Cyclophosphamide 300 mg/m2 every 12 hours for a total dose of 1800 mg/m2.	II	17	15 (88%) CR or PR

**Kleinerman ES, et al **J. Clin. Oncol. 10, 1310-6. [57]	1992	L-MTP-PE 2 mg/m^2 ^infused during a 1-h period twice a wk for 12 wks, then once a wk for 12 wks.	II	16	Not mentioned

**Kleinerman ES, et al **Am J Clin Oncol. 18(2):93-9. [58]	1995	L-MTP-PE, 2 mg/m^2^, i.v over a 1-h twice a wk for 12 wks in 12 pts (Group 1). 16 pts (Group 2) had 2 mg/m^2 ^L-MTP-PE twice a wk for 12 wks, then once a wk for 12 wks, for a total of 24 wks.	II	36	2 (6%) SD

**Kleinerman ES, et al **J. Immunother. Emphasis Tumor Immunol., 17, 181-93. [59]	1995	IFO 1.8 g/m^2 ^for 5 days every 21 days for up to 8 cycles. L-MTP-PE twice weekly for 12 weeks, then once weekly for 12 weeks	IIb	9	Not mentioned

**Harris MB**, **et al **Med Ped Oncol 24, 87-92. [60]	1995	Two courses of ifosfamide (2400 mg/m^2 ^× 5 days) administered 3 wks apart	II	33	Stratum 1: 1 (11%) CR, 8 (24%) PR Stratum 2: 1 (3%) CR, 2 (7%) PR

**Patel SR, et al **Cancer; 78:741-4. [61]	1996	Paclitaxel 175 mg/m^2 ^24-h i.v	II	15	0% OR

**Gentet JC**, et al Eur J Cancer, Vol. 33, No. 2, 232-7 [62]	1997	Two courses of IFO 3 g/m^2^/day and etoposide 75 mg/m^2^/day for 4 days.	II	27	6 (23%) CR, 7 (25%) PR, 5 (23%) SD

**Worth LL, et al **Clin Cancer Res. 3(10):1721-9. [63]	1997	IL-1alpha followed by ICE daily for 5 days/3 wks.	II	9	3 (34%) PR, 1 (11%) SD

**Voûte PA, et al **Annals of Oncology 10: 1211-8. [64]	1999	IFO 3 g/m^2^/dl-2, DOX 25 mg/m^2^/dl-3 i.v. bolus and CDDP 100 mg/m^2^/dl.	II	103	5 year survival was 62% in limb-non-metastatic, 41% in axial skeletal and 16% in limb metastatic patients.

**Fagioli F, et al **Journal of Clinical Oncology, Vol 20, Issue 8:2150-6 [65]	2002	High-dose chemotherapy consisted of carboplatin and etoposide followed by stem-cell rescue.	I	32	25 (78%) CR, 6 (19%) PD, 3 year OS 20% 3 year DFS 12%

**Laverdiere C, et al **Cancer; 98:832-40. [66]	2003	Trabectedin 1500 micro g/m^2 ^as a 24-h i.v every 3 wks.	II	25	0% OR

**McTiernan A, et al**. Sarcoma, Vol. 8, No. 2/3, 71-6. [67]	2004	Docetaxel 100 mg/m^2 ^1-h i.v every 3 wks	II	14	1 (7%) PR, 2 (14%) SD

**Ferrari S et al **J Clin Oncol 23:8845-52. [68]	2005	Two blocks of high-dose IFO (15 g/m^2^), MTX (12 g/m^2^), CDDP (120 m g/m^2^), and DOX (75 m g/m^2^)	II	182	5-year EFS 64% and OS 77%

**Arpaci F, et al **Cancer; 104:1058-65. [69]	2005	2 cycles of CDDP, DOX, and IFO followed by HDC and APBSCT.	II	22	3 year: OS was 83% & 70% DFS

**McTiernan A, et al **Pediatr Blood Cancer. 46(3):345-50. [70]	2006	IFO 2.5 g/m^2 ^etoposide 150 mg/m^2 ^and DOX 20 mg/m^2 ^on days 1-3, every 21 days, with interval MTX 12 g/m^2 ^given on day 14, for a maximum of 8 cycles.	I/II	13	0% OR

**Seibel NL, et al **Cancer 2007; 109:1646-53. [71]	2007	Topotecan daily x5 followed by chemotherapy (IFO, carboplatin, ICE, alternating with CDDP and CD.	I	28	1 (6.6%) PR, 1 (6.6%) CLR 2- and 5-year EFS rates 7% & 4%, resp, 2- and 5-year OS rates 44% and 22%, resp.

**Basaran M, et al **Oncology;72:255-60 [72]	2007	Epirubicin 90 mg/m^2 ^cisplatin 100 mg/m^2 ^on day 1 and IFO 2.0 g/m^2 ^day with an equivalent dose of mesna on days 2-4, repeated every 21 days	II	38	10 (26%) CR, 5 year DFS 41.9%, OS 48.2%.

**Iwamoto Y, et al **J Orthop Sci 14:397-04. [73]	2009	Preoperative chemotherapy: HD-MTX, CDDP, and ADR.	II	113	5 year OS 77.9% 5 year EFS 65.5%

**Berger M, et al **Cancer 115: 2980 -7. [74]	2009	Cyclophosphamide 4 g/m^2 ^on Day 1 followed by etoposide at 200 mg/m^2 ^on Days 2, 3, and 4.	II	26	9 (35%) SD, OS at 1 yr 50%, PFS 42%

**Chawla S P, et al **Mol Ther. 17:1651-7. [51]	2009	Escalating doses of Rexin-G i.v from 8 × 10^11 ^to 24 × 10^11 ^colony forming units (cfu)/cycle.	II I/II	Osteo = 22 Sar = 20 Osteo = 0	10 (58.8%) SD 3 (50%) SD(lowest dose) 10 (71%) SD(higher dose)

*Abbreviations- *ADR = adriamycin; APBSCT = autologous peripheral blood stem cell support transplantation; CDDP = cisplatin; Cfu = colony forming units; CR = complete response; DOX = doxorubicin; EFS = event free survival; GCSF = Granulocyte colony-stimulating factor; HD-MTX = High-dose Methotrexate; ICE = etoposide; IFO = ifosfamide; IL-6 = interleukin -6; IL-8 = interleukin-8; L-MTP-PE = liposomal muramyl tripeptide phosphatidyl ethanolamine; MTX = methotrexate; OR = objective response; PD = progressive disease; PFS = progression free survival; OS = overall survival; RDI = relative dose intensity; resp = respectively; Sar = sarcoma; TNF = tumour necrosis factor

**Table 4:** Characteristics of eligible trials for Ewing sarcoma only

Author	Year	Intervention	Phase	No of Patients	Outcome
**Hawkins et al**, Med Pediatric Oncol, 34 (5): 328-37 [33]	2000	Myeloablative therapy followed by HSCT.	I	16	3-year EFS 36%

**Kolb et al**, J Clin Oncol, 21 (18): 3423-30 [75]	2003	7 cycles of chemotherapy consisted of Cyclo, Doxo, Vin, Ifos and Etop	II	68	29 (43%) CR, 13 (19%) PD or SD

**Meyer et al**, Sarcoma, 7 (1): 13-7 [76]	2003	Doc thrice weekly for a maximum of six cycles.	II	14	1 (14%) PR, 2 (28%) SD

**Bernstein et al**, J Clin Oncol, 24 (1): 152-9 [77]	2006	High-dose induction therapy followed by window period. Randomization between topo, topo + cyclo or no treatment. No window treatment patients received Amifostine	II	117	45 (43%) CR, 41 (39%) PR, 14 (14%) SD

**Womer et al**, ASCO abstract	2008	Vin, Doxo and Cyclo alternating with Ifos and Etop, for 14 cycles. Regimen A: 3 weeks cycle, regimen B: 2 week cycle. Primary tumor treatment was scheduled to begin week 13.	II	587	EFS at a median of 3 years was 65% in Regimen A and 76% in Regimen B.

**Rosenthal et al**, Bone Marrow Transplantation, 42: 311-8 [78]	2008	HDT followed by HSCT.	I	22	3-year EFS 47%, 3-year OS 45%

**DuBois et al**, Pediatric Blood Cancer, 52 (3): 324-7 [79]	2009	Cytarabine.	II	10	1 (10%) SD

**Casey et al**, Pediatric Blood Cancer, 53 (6): 1029-34 [80]	2009	Irinotecan and Temozolomide.	II	20	5 (25%) CR, 7 (36%) PR

**Olmos et al**, Lancet Oncol, 11: 129-35 [56]	2010	A subgroup of Ewing Sarcoma patients were treated with Figitumumab	I	15	1 (6%) CR, 1 (6%) PR, 6 (37%) SD

*Abbreviations*: CR = complete response; Cyclo = cyclophosphamide; Doc = docetaxel; Doxo = doxorubucine; EFS = event free survival; Etop = etoposide; G-CSF = granulocyte-colony stimulating factor; HDT = high-dose chemotherapy; HSCT = hematopoietic stem cell transplantation; Ifos = ifosfamide; OR = overall response; OS = overall survival; PD = progressive disease; PR = partial response; SD = stabile disease; Topo = topotecan; Vin = vincristine

For the number of patients enrolled in the eligible trials the results were disappointing (Figure 2B), with an almost stable number of patients in osteosarcoma trials, except for 1999, 2005 and 2009 when the results from larger phase II trials were published (Table 3). For Ewing sarcoma there were increasing numbers of patients in early phase trials, especially in 2008 when the result of the largest trial in Ewing sarcoma patients was published (Table 4). Out of all the 780 osteosarcoma patients enrolled in phase I/II trials including only osteosarcoma patients, 762 were evaluable and 58 patients (8%) achieved complete response (CR), 21 (2.8%) showed partial response (PR) and 30 (4%) developed stable disease (SD). For the trials enrolling only Ewing sarcoma patients, 869 were recruited and 840 were evaluable, 80 had CR (9.5%), 63 (7.5%) showed PR and 23 (3%) developed SD.

Looking at the trials subdivided for chemotherapy or biological treatment (Tables 1 and 2) in the past twenty years, there seems to have been a shift towards biologically based treatments instead of chemotherapy. Even so most of the phase III trials are still chemotherapy based treatments.

For current ongoing phase I and phase II studies we found 156 trials that are open and recruiting sarcoma patients. These trials recruit sarcoma patients in general or patients with solid tumours or soft tissue sarcoma. Studies enrolling only osteosarcoma or Ewing sarcoma patients are rare. For osteosarcoma we found four trials that are recruiting patients and two that are already active but not yet recruiting patients (Table 5). Of the trials two were testing chemotherapy treatment, three biologically agents and one a combination of both. For Ewing sarcoma we found no trials that enrol only Ewing patients and two that are still active but not currently enrolling patients.

**Table 5:** Ongoing phase I or II trials in osteosarcoma (Assessed from www.ClinicalTrials.gov)

ClinicalTrials.gov Identifier	Sponsors	Title	Status
NCT00102531	Transave	Phase Ib/IIa non-randomised study of SLIT Cisplatin by inhalation in the treatment of patients with relapsed/progressive osteosarcoma metastatic to the lung	Recruiting

NCT01002092	Shandong Simcere-Medgenn Bio-pharmaceutical Co., Ltd	A randomized, controlled multicenter trial of Endostar combined with chemotherapy for treatment of osteosarcoma (phase II)	Recruiting

NCT00889057	Italian Sarcoma Group	Phase II, open label, non-randomized study of second or third line treatment with Sorafenib (BAY 43-9006) in patients affected by relapsed high-grade osteosarcoma	Recruiting

NCT00752206 NCT00923286	Sarcoma Alliance for Research through Collaboration (SARC)	A randomized, double-blinded, placebo-controlled, multi-institutional, phase II study of AZD0530, a selective Src kinase inhibitor, in patients with recurrent osteosarcoma localized to the lung	Recruiting

NCT01005043	University of Heidelberg	Phase I/II therapy non-randomised trial to determine the safety and efficacy of heavy ion radiotherapy in patients with osteosarcoma	Not yet recruiting

NCT00902044	Baylor College of Medicine	Administration of Her2 Chimeric antigen receptor expressing T Cells for subjects with advanced osteosarcoma (HEROS) (phase I)	Not yet recruiting

When searching for studies that are enrolling sarcoma patients we found a number of trials that were preliminary stopped. For example SARC011, this is a phase II trial of R1507, a recombinant human monoclonal antibody to the Insulin-like growth factor-1 receptor for the treatment of patients with recurrent or refractory Ewing sarcoma, osteosarcoma, synovial sarcoma, rhabdomyosarcoma and other sarcomas. In December 2009, Roche/Genentech decided to discontinue the development of their IGF-1R antibody although the drug had shown important clinical benefit http://www.cancer.gov/ncicancerbulletin/111610/page5. Roche said the decision was due to the available clinical data, the large number of molecules targeting the same pathway that are presently in development and the prioritization of the Roche portfolio. The decision was not as a result of safety concerns. Both this study, and a Phase I/II with figitumumab (Pfizer) have been published since completing this survey, and show low but durable response rates in a subset of relapsed Ewing patients (10-20%) [17,18].

Bone sarcoma is fatal in approximately one third of the children and young adults in whom it is diagnosed. This mortality rate has not changed greatly in the two decades since the initial introduction of surgery and chemotherapy. The primary cause of death in treated patients is recurrent metastatic disease often to the lung. In the group of patients who present either with metastatic or relapsed disease, it is especially important to conduct biologically based trials with small groups of patients from which quantitative and quick answers of whether a treatment intervention is either working or not can be obtained. Importantly, tumour material obtained may provide informative clues for future studies especially if analysed with genomic technologies.

It is difficult to do trials in an orphan disease, mainly because of the problems of recruiting enough eligible patients for the trial. In our literature search, we identified 42 phase I/II trials for sarcoma patients which were published between 1990 and 2010. However the phase I trials for general sarcoma (Table 1) are not complete, this is because or our search criteria. With a PubMed search you only search the abstract of the article, so if sarcoma is not mentioned there we didn't find the article. In phase I trials quite a few different tumour types are sometimes enrolled and only the one who show results are mentioned in the abstract. Some of these articles we found in the Cochrane Controlled Trial Register or the ClinicalTrials.gov database, but not all trials are registered in these databases. Except with a very wide PubMed search using (phase 1) as only search criteria we don't think that it is possible to find all phase I trials enrolling sarcoma patients.

Of the trials we did found with our search criteria twenty-one were phase I, two phase I/II and nineteen phase II trials. Only eight clinical trials included only Ewing sarcoma patients; of this group two were phase I and six were phase II trials. We found only twenty trials which included only osteosarcoma patients. There were two phase I, sixteen phase II, and two phase I/II trials. A total of 3,736 patients were included in the clinical trials, of which 1,263 were Ewing sarcoma and 1,114 were osteosarcoma patients. From the identified trials, the results are not convincing for benefit and most of the time even disappointing, from osteosarcoma trials we found 8% CR, 2.8% PR and 4% SD. For Ewing sarcoma the results are 9.5% CR, 7.5% PR and 3% SD. And if the drugs seem to be effective, a lot of toxicity is reported (Tables 1, 2, 3, 4). Looking at the number of trials we found for sarcoma patients, the results seem encouraging as a high number of patients were included. However, if we make a calculation of the number of new Ewing sarcoma patients expected in the last twenty years, assuming a world population of five billion and an incidence of 2 in 1.000,000, approximately 200,000 new cases would be expected in twenty years world-wide. Of these patients, we could only find 1,263 reported and included in published clinical trials. For osteosarcoma the number is even worse, with 500,000 new cases expected in twenty years and only 1,114 reported in trials.

To improve these results changes in conducting trials for orphan state diseases have to be made. It is important to collaborate between nations; the Sarcoma Alliance for Research through Collaboration (SARC) is a good example based principally in North America. SARC is a non-profit organization started in 2003. Funding is provided by generous donations, sponsors and grants. SARC works with healthcare professionals as part of a collaborative multidisciplinary team from over 35 institutions in the USA. It works with a number of international institutions and provides the infrastructure for collaboration between physicians, researchers, and medical institutions from across the worlds who are engaged in clinical research for development of new standards of sarcoma patient treatment, education, and prevention. This organization facilitates dialogue and collaboration among sarcoma researchers and clinicians, assists in the development and dissemination of protocols for clinical trials and information relating to sarcoma research and the results of clinical trials, administers research grants and funding for clinical trials, and acts as a primary resource for those treating patients with sarcoma. The EORTC Soft Tissue and Bone Sarcoma Group is one of SARC's European counterpart, conducting multicenter academic studies in sarcoma, including some early stage translational studies, although it's main contribution in the past has been in the field of larger phase III trials soft tissue sarcoma. The European Osteosarcoma Intergroup, EURAMOS and EuroEwing consortium are examples of groups acting primarily as platforms for phase III studies in bone sarcomas.

The EC-granted European network, EuroBoNeT http://www.eurobonet.eu, was the first central organized network of excellence for integrated bone sarcoma research and staff exchange, in order to increase and disseminate knowledge of primary bone tumours at the molecular level for development of new tools for patient care. This integration, exchange of material (virtual BioBank), standard operating protocols and the use of technology platforms enabled researchers to obtain statistically significant datasets, otherwise not achievable due to the rareness of the condition and the large number of sub-entities. Without these kinds of collaborations, the development of new treatment strategies in osteosarcoma and Ewing sarcoma is going to be very difficult. But perhaps most importantly is that phase I/II clinical trials have to be conducted. To achieve this funding needs to be established from industry and governments to feed through these collaborative structures over time frames that might result in enduring progress. Recent progress has also been made with the FP7 funding of EuroSarc, European trials in rare sarcomas within an integrated translational trial network (2011-2016). In this new translational trial network, the challenges of combining high quality disease research into clinical trials in being addressed, and progresses the field beyond conventional trials and the existing clinical trial networks.

In addition to the challenge of identifying the most promising agents for clinical trials in bone sarcoma, obstacles inherent to this disease further complicate the successful design and completion of trials of novel agents. In evaluating the efficacy of novel agents, the standard approach is to use objective response criteria, such as Response Evaluation Criteria in Solid Tumours (RECIST) [19], to compare the size and/or volume of lesions pre-treatment and at regular intervals during and post-treatment. New methods for tumour response which may be more precise are radiological response based on new criteria, e.g. ^18^FDG-PET-CT and contrast enhanced MRI. For a patient to be eligible for a trial using this approach, he or she must have measurable disease. The traditional approach for patients with relapsed bone sarcoma, in particular pulmonary relapse, has been surgical resection, which at least temporarily renders a patient free of radiographic measurable disease and thus, ineligible for most trials of novel agents. Unfortunately novel targeted therapies may be most effective in the setting of minimal residual disease. The use of creative surrogate end points to estimate response and perhaps render the combination of surgical resection and treatment on trials of new agents not mutually exclusive would allow for better evaluation of new therapies in patients with bone sarcoma [20].

When conducting the literature search we found that the results of many trials are not published. We can only guess what the reason for this is, but is seems logical that the trial results were disappointing. From the phase I/II trials only a very limited number of treatments proceeded to phase III trial. For Ewing sarcoma we found three clinical trials conducted in the last twenty years and two trials which are being recruiting at this moment and for osteosarcoma we found twenty-two phase III clinical trials and four active trials currently recruiting patients and one is ongoing trial but not recruiting.

Most of the phase I/II trials that have been conducted recruited patients with general sarcoma or solid tumours. In these trials it is very difficult to say anything about disease specific response and it is therefore almost impossible to translate the results to clinical practise and patients with a specific sort of cancer. Of course it is understandable why researchers test a drug in different patient populations in the same trial, especially is rare cancers like bone sarcoma, but it makes it more difficult to proceed to a disease specific phase II or even phase III trial.

While the current standard of care continues to be multi-agent chemotherapy and surgical resection, it is clear that further intensification of traditional chemotherapy regimens is limited by toxicity, and new approaches are needed. Some promising novel agents are currently in or are entering into phase II trials in children with relapsed bone sarcoma. For example, Muramyl Tripeptide Phosphatidylethanolamine (MTP-PE) is the first drug to be approved for osteosarcoma in Europe for ten years [21]. In Ewing sarcoma the identification of the Insulin-like Growth Factor 1 Receptor (IGF1R) pathway deregulation, as a consequence of the EWS-FLI1 translocation seems to be a new treatment strategy to be explored. The incorporation of these new treatments into the up-front therapy for bone sarcoma in the near future holds promise for improving outcomes.

## Lists of abbreviations

EMEA: European Medicines (Evaluation) Agency; EFS: Event free survival; IGF1R: Insulin-like Growth Factor 1 Receptor; MTP-PE: Muramyl Tripeptide Phosphatidylethanolamine; OS: Overall survival; PET: Positron emission tomography; PFS: Progression free survival; RECIST: Response Evaluation Criteria in Solid Tumours; SARC: Sarcoma Alliance for Research through Collaboration network.

## Competing interests

All authors have read and approved the final manuscript, and have no competing financial interests in the publication of this manuscript. No organisation is funding or implicated in the manuscripts analysis and interpretation. Academic interests of the authors are to improve the outcome of patients with sarcoma, and this publication forms part of the deliverable output from EU FP7 funding from EuroBoNeT.

## Authors' contributions

ABH conceived the study. AVM and AP collected data with ABH. Data extraction was conducted independently by two authors (AB and AVM). Differences in data extraction were resolved by consensus with a third author (ABH). AVM and ABH wrote the paper and AP, PC and AG made comments. All authors have read and approved the final manuscript.

## References

[B1] Mohseny AB Szuhai K Romeo S Buddingh EP Briaire-de Bruijn I de Jong D van Pel M Cleton-Jansen AM Hogendoorn PC Osteosarcoma originates from mesenchymal stem cells in consequence of aneuploidization and genomic loss of Cdkn2 J Pathol 2009 219 294 305 10.1002/path.2603 19718709

[B2] Gatta G van der Zwan JM Casali PG Siesling S Dei Tos AP Kunkler I Otter R Licitra L Mallone S Tavilla A Trama A Capocaccia R Rare cancers are not so rare: The rare cancer burden in Europe Eur J Cancer 2011 47 2493 2511 10.1016/j.ejca.2011.08.008 22033323

[B3] Bacci G Fabbri N Balladelli A Forni C Palmerini E Picci P Treatment and prognosis for synchronous multifocal osteosarcoma in 42 patients J Bone Joint Surg Br 2006 88 1071 1075 16877608 10.1302/0301-620X.88B8.17809

[B4] Lewis IJ Nooij MA Whelan J Sydes MR Grimer R Hogendoorn PC Memon MA Weeden S Uscinska BM van Glabbeke M Kirkpatrick A Hauben EI Craft AW Taminiau AH Improvement in histologic response but not survival in osteosarcoma patients treated with intensified chemotherapy: a randomized phase III trial of the European Osteosarcoma Intergroup J Natl Cancer Inst 2007 99 112 128 10.1093/jnci/djk015 17227995

[B5] Chou AJ Kleinerman ES Krailo MD Chen Z Betcher DL Healey JH Conrad EU Nieder ML Weiner MA Wells RJ Womer RB Meyers PA Addition of muramyl tripeptide to chemotherapy for patients with newly diagnosed metastatic osteosarcoma: a report from the Children's Oncology Group Cancer 2009 115 5339 5348 10.1002/cncr.24566 19637348 PMC2783515

[B6] Eilber F Giuliano A Eckardt J Patterson K Moseley S Goodnight J Adjuvant chemotherapy for osteosarcoma: a randomized prospective trial J Clin Oncol 1987 5 21 26 3543236 10.1200/JCO.1987.5.1.21

[B7] Goorin AM Perez-Atayde A Gebhardt M Andersen JW Wilkinson RH Delorey MJ Watts H Link M Jaffe N Frei E Weekly high-dose methotrexate and doxorubicin for osteosarcoma: the Dana-Farber Cancer Institute/the Children's Hospital--study III J Clin Oncol 1987 5 1178 1184 3476688 10.1200/JCO.1987.5.8.1178

[B8] Link MP Goorin AM Miser AW Green AA Pratt CB Belasco JB Pritchard J Malpas JS Baker AR Kirkpatrick JA The effect of adjuvant chemotherapy on relapse-free survival in patients with osteosarcoma of the extremity N Engl J Med 1986 314 1600 1606 10.1056/NEJM198606193142502 3520317

[B9] Buddingh EP Anninga JK Versteegh MI Taminiau AH Egeler RM van Rijswijk CS Hogendoorn PC Lankester AC Gelderblom H Prognostic factors in pulmonary metastasized high-grade osteosarcoma Pediatr Blood Cancer 2010 54 216 221 19890902 10.1002/pbc.22293

[B10] Riggi N Stamenkovic I The Biology of Ewing sarcoma Cancer Lett 2007 254 1 10 10.1016/j.canlet.2006.12.009 17250957

[B11] Szuhai K Ijszenga M de Jong D Karseladze A Tanke HJ Hogendoorn PC The NFATc2 gene is involved in a novel cloned translocation in a Ewing sarcoma variant that couples its function in immunology to oncology Clin Cancer Res 2009 15 2259 2268 10.1158/1078-0432.CCR-08-2184 19318479

[B12] de Alava E Lozano MD Patino A Sierrasesumaga L Pardo-Mindan FJ Ewing family tumors: potential prognostic value of reverse-transcriptase polymerase chain reaction detection of minimal residual disease in peripheral blood samples Diagn Mol Pathol 1998 7 152 157 10.1097/00019606-199806000-00005 9836070

[B13] Le Deley MC Delattre O Schaefer KL Burchill SA Koehler G Hogendoorn PC Lion T Poremba C Marandet J Ballet S Pierron G Brownhill SC Nesslbock M Ranft A Dirksen U Oberlin O Lewis IJ Craft AW Jurgens H Kovar H Impact of EWS-ETS fusion type on disease progression in Ewing's sarcoma/peripheral primitive neuroectodermal tumor: prospective results from the cooperative Euro-E.W.I.N.G. 99 trial J Clin Oncol 2010 28 1982 1988 10.1200/JCO.2009.23.3585 20308673

[B14] Rodriguez-Galindo C Navid F Liu T Billups CA Rao BN Krasin MJ Prognostic factors for local and distant control in Ewing sarcoma family of tumors Ann Oncol 2008 19 814 820 17998282 10.1093/annonc/mdm521

[B15] Bernstein ML Devidas M Lafreniere D Souid AK Meyers PA Gebhardt M Stine K Nicholas R Perlman EJ Dubowy R Wainer IW Dickman PS Link MP Goorin A Grier HE Intensive therapy with growth factor support for patients with Ewing tumor metastatic at diagnosis: Pediatric Oncology Group/Children's Cancer Group Phase II Study 9457--a report from the Children's Oncology Group J Clin Oncol 2006 24 152 159 10.1200/JCO.2005.02.1717 16382125

[B16] Hamada C The role of meta-analysis in cancer clinical trials Int J Clin Oncol 2009 14 90 94 10.1007/s10147-008-0876-x 19390938

[B17] Juergens H Daw NC Geoerger B Ferrari S Villarroel M Aerts I Whelan J Dirksen U Hixon ML Yin D Wang T Green S Paccagnella L Gualberto A Preliminary efficacy of the anti-insulin-like growth factor type 1 receptor antibody figitumumab in patients with refractory ewing sarcoma J Clin Oncol 2011 29 4534 4540 10.1200/JCO.2010.33.0670 22025154 PMC3236653

[B18] Pappo AS Patel SR Crowley J Reinke DK Kuenkele KP Chawla SP Toner GC Maki RG Meyers PA Chugh R Ganjoo KN Schuetze SM Juergens H Leahy MG Geoerger B Benjamin RS Helman LJ Baker LH R1507, a Monoclonal Antibody to the Insulin-Like Growth Factor 1 Receptor, in Patients With Recurrent or Refractory Ewing Sarcoma Family of Tumors: Results of a Phase II Sarcoma Alliance for Research Through Collaboration Study J Clin Oncol 2011 29 4541 4547 10.1200/JCO.2010.34.0000 22025149 PMC3236654

[B19] Eisenhauer EA Therasse P Bogaerts J Schwartz LH Sargent D Ford R Dancey J Arbuck S Gwyther S Mooney M Rubinstein L Shankar L Dodd L Kaplan R Lacombe D Verweij J New response evaluation criteria in solid tumours: revised RECIST guideline (version 1.1) Eur J Cancer 2009 45 228 247 10.1016/j.ejca.2008.10.026 19097774

[B20] Le Cesne A Van Glabbeke M Verweij J Casali PG Findlay M Reichardt P Issels R Judson I Schoffski P Leyvraz S Bui B Hogendoorn PC Sciot R Blay JY Absence of progression as assessed by response evaluation criteria in solid tumors predicts survival in advanced GI stromal tumors treated with imatinib mesylate: the intergroup EORTC-ISG-AGITG phase III trial J Clin Oncol 2009 27 3969 3974 10.1200/JCO.2008.21.3330 19620483 PMC2799153

[B21] Meyers PA Muramyl tripeptide (mifamurtide) for the treatment of osteosarcoma Expert Rev Anticancer Ther 2009 9 1035 1049 10.1586/era.09.69 19671023

[B22] Pratt CB Meyer WH Howlett N Douglass EC Bowman LC Poe D Mounce K Kun LE Houghton JA Phase II study of 5-fluorouracil/leucovorin for pediatric patients with malignant solid tumors Cancer 1994 74 2593 2598 10.1002/1097-0142(19941101)74:9<2593::AID-CNCR2820740930>3.0.CO;2-C 7923016

[B23] Kushner BH Meyers PA Gerald WL Healey JH La Quaglia MP Boland P Wollner N Casper ES Aledo A Heller G Very-high-dose short-term chemotherapy for poor-risk peripheral primitive neuroectodermal tumors, including Ewing's sarcoma, in children and young adults Journal of clinical oncology: official journal of the American Society of Clinical Oncology 1995 13 2796 2804 7595741 10.1200/JCO.1995.13.11.2796

[B24] Antman K Crowley J Balcerzak SP Kempf RA Weiss RB Clamon GH Baker LH A Southwest Oncology Group and Cancer and Leukemia Group B phase II study of doxorubicin, dacarbazine, ifosfamide, and mesna in adults with advanced osteosarcoma, Ewing's sarcoma, and rhabdomyosarcoma Cancer 1998 82 1288 1295 10.1002/(SICI)1097-0142(19980401)82:7<1288::AID-CNCR11>3.0.CO;2-2 9529020

[B25] Blaney SM Needle MN Gillespie A Sato JK Reaman GH Berg SL Adamson PC Krailo MD Bleyer WA Poplack DG Balis FM Phase II trial of topotecan administered as 72-hour continuous infusion in children with refractory solid tumors: a collaborative Pediatric Branch, National Cancer Institute, and Children's Cancer Group Study Clinical cancer research: an official journal of the American Association for Cancer Research 1998 4 357 360 9516923

[B26] Lucidarme N Valteau-Couanet D Oberlin O Couanet D Kalifa C Beaujean F Lapierre V Hartmann O Phase II study of high-dose thiotepa and hematopoietic stem cell transplantation in children with solid tumors Bone marrow transplantation 1998 22 535 540 10.1038/sj.bmt.1701395 9758339

[B27] Berg SL Blaney SM Sullivan J Bernstein M Dubowy R Harris MB Phase II trial of pyrazoloacridine in children with solid tumors: a Pediatric Oncology Group phase II study Journal of pediatric hematology/oncology 2000 22 506 509 10.1097/00043426-200011000-00006 11132217 PMC4008246

[B28] Delaloge S Yovine A Taamma A Riofrio M Brain E Raymond E Cottu P Goldwasser F Jimeno J Misset JL Marty M Cvitkovic E Ecteinascidin-743: a marine-derived compound in advanced, pretreated sarcoma patients--preliminary evidence of activity Journal of clinical oncology: official journal of the American Society of Clinical Oncology 2001 19 1248 1255 11230465 10.1200/JCO.2001.19.5.1248

[B29] Saylors RL Stine KC Sullivan J Kepner JL Wall DA Bernstein ML Harris MB Hayashi R Vietti TJ Cyclophosphamide plus topotecan in children with recurrent or refractory solid tumors: a Pediatric Oncology Group phase II study Journal of clinical oncology: official journal of the American Society of Clinical Oncology 2001 19 3463 3469 11481351 10.1200/JCO.2001.19.15.3463

[B30] Wagner LM Crews KR Iacono LC Houghton PJ Fuller CE McCarville MB Goldsby RE Albritton K Stewart CF Santana VM Phase I trial of temozolomide and protracted irinotecan in pediatric patients with refractory solid tumors Clinical cancer research: an official journal of the American Association for Cancer Research 2004 10 840 848 10.1158/1078-0432.CCR-03-0175 14871959

[B31] Lau L Supko JG Blaney S Hershon L Seibel N Krailo M Qu W Malkin D Jimeno J Bernstein M Baruchel S A phase I and pharmacokinetic study of ecteinascidin-743 (Yondelis) in children with refractory solid tumors. A Children's Oncology Group study Clinical cancer research: an official journal of the American Association for Cancer Research 2005 11 672 677 15701855

[B32] Van Winkle P Angiolillo A Krailo M Cheung YK Anderson B Davenport V Reaman G Cairo MS Ifosfamide, carboplatin, and etoposide (ICE) reinduction chemotherapy in a large cohort of children and adolescents with recurrent/refractory sarcoma: the Children's Cancer Group (CCG) experience Pediatric blood & cancer 2005 44 338 347 10.1002/pbc.20227 15503297

[B33] Hawkins D Barnett T Bensinger W Gooley T Sanders J Busulfan, melphalan, and thiotepa with or without total marrow irradiation with hematopoietic stem cell rescue for poor-risk Ewing-Sarcoma-Family tumors Medical and pediatric oncology 2000 34 328 337 10.1002/(SICI)1096-911X(200005)34:5<328::AID-MPO3>3.0.CO;2-4 10797354

[B34] Navid F Santana VM Billups CA Merchant TE Furman WL Spunt SL Cain AM Rao BN Hale GA Pappo AS Concomitant administration of vincristine, doxorubicin, cyclophosphamide, ifosfamide, and etoposide for high-risk sarcomas: the St. Jude Children's Research Hospital experience Cancer 2006 106 1846 1856 10.1002/cncr.21810 16541446

[B35] Wagner-Bohn A Henze G von Stackelberg A Boos J Phase II study of gemcitabine in children with relapsed leukemia Pediatric blood & cancer 2006 46 262 10.1002/pbc.20632 16331664

[B36] Zaucha RE Buckner DC Barnett T Holmberg LA Gooley T Hooper HA Maloney DG Appelbaum F Bensinger WI Modified total body irradiation as a planned second high-dose therapy with stem cell infusion for patients with bone-based malignancies International journal of radiation oncology, biology, physics 2006 64 227 234 10.1016/j.ijrobp.2005.06.005 16169680

[B37] Zwerdling T Krailo M Monteleone P Byrd R Sato J Dunaway R Seibel N Chen Z Strain J Reaman G Phase II investigation of docetaxel in pediatric patients with recurrent solid tumors: a report from the Children's Oncology Group Cancer 2006 106 1821 1828 10.1002/cncr.21779 16532433

[B38] Geoerger B Doz F Gentet JC Mayer M Landman-Parker J Pichon F Chastagner P Rubie H Frappaz D Le Bouil A Gupta S Vassal G Phase I study of weekly oxaliplatin in relapsed or refractory pediatric solid malignancies Journal of clinical oncology: official journal of the American Society of Clinical Oncology 2008 26 4394 4400 10.1200/JCO.2008.16.7585 18802151

[B39] McGregor LM Spunt SL Furman WL Stewart CF Schaiquevich P Krailo MD Speights R Ivy P Adamson PC Blaney SM Phase 1 study of oxaliplatin and irinotecan in pediatric patients with refractory solid tumors: a children's oncology group study Cancer 2009 115 1765 1775 10.1002/cncr.24175 19170226 PMC2897817

[B40] Daw NC Furman WL Stewart CF Iacono LC Krailo M Bernstein ML Dancey JE Speights RA Blaney SM Croop JM Reaman GH Adamson PC Phase I and pharmacokinetic study of gefitinib in children with refractory solid tumors: a Children's Oncology Group Study Journal of clinical oncology: official journal of the American Society of Clinical Oncology 2005 23 6172 6180 10.1200/JCO.2005.11.429 16135484

[B41] Maki RG Kraft AS Scheu K Yamada J Wadler S Antonescu CR Wright JJ Schwartz GK A multicenter Phase II study of bortezomib in recurrent or metastatic sarcomas Cancer 2005 103 1431 1438 10.1002/cncr.20968 15739208

[B42] Villablanca JG Krailo MD Ames MM Reid JM Reaman GH Reynolds CP Phase I trial of oral fenretinide in children with high-risk solid tumors: a report from the Children's Oncology Group (CCG 09709) Journal of clinical oncology: official journal of the American Society of Clinical Oncology 2006 24 3423 3430 10.1200/JCO.2005.03.9271 16849757

[B43] Bagatell R Gore L Egorin MJ Ho R Heller G Boucher N Zuhowski EG Whitlock JA Hunger SP Narendran A Katzenstein HM Arceci RJ Boklan J Herzog CE Whitesell L Ivy SP Trippett TM Phase I pharmacokinetic and pharmacodynamic study of 17-N-allylamino-17-demethoxygeldanamycin in pediatric patients with recurrent or refractory solid tumors: a pediatric oncology experimental therapeutics investigators consortium study Clinical cancer research: an official journal of the American Association for Cancer Research 2007 13 1783 1788 10.1158/1078-0432.CCR-06-1892 17363533

[B44] Jimeno A Daw NC Amador ML Cusatis G Kulesza P Krailo M Ingle AM Blaney SM Adamson P Hidalgo M Analysis of biologic surrogate markers from a Children's Oncology Group Phase I trial of gefitinib in pediatric patients with solid tumors Pediatric blood & cancer 2007 49 352 357 10.1002/pbc.20753 16425266

[B45] Kramer K Humm JL Souweidane MM Zanzonico PB Dunkel IJ Gerald WL Khakoo Y Yeh SD Yeung HW Finn RD Wolden SL Larson SM Cheung NK Phase I study of targeted radioimmunotherapy for leptomeningeal cancers using intra-Ommaya 131-I-3F8 Journal of clinical oncology: official journal of the American Society of Clinical Oncology 2007 25 5465 5470 10.1200/JCO.2007.11.1807 18048828

[B46] Bond M Bernstein ML Pappo A Schultz KR Krailo M Blaney SM Adamson PC A phase II study of imatinib mesylate in children with refractory or relapsed solid tumors: a Children's Oncology Group study Pediatric blood & cancer 2008 50 254 258 10.1002/pbc.21132 17262795

[B47] Fox E Maris JM Widemann BC Goodspeed W Goodwin A Kromplewski M Fouts ME Medina D Cohn SL Krivoshik A Hagey AE Adamson PC Balis FM A phase I study of ABT-751, an orally bioavailable tubulin inhibitor, administered daily for 21 days every 28 days in pediatric patients with solid tumors Clinical cancer research: an official journal of the American Association for Cancer Research 2008 14 1111 1115 10.1158/1078-0432.CCR-07-4097 18281544

[B48] Jakacki RI Hamilton M Gilbertson RJ Blaney SM Tersak J Krailo MD Ingle AM Voss SD Dancey JE Adamson PC Pediatric phase I and pharmacokinetic study of erlotinib followed by the combination of erlotinib and temozolomide: a Children's Oncology Group Phase I Consortium Study Journal of clinical oncology: official journal of the American Society of Clinical Oncology 2008 26 4921 4927 10.1200/JCO.2007.15.2306 18794549 PMC2652086

[B49] Langevin AM Bernstein M Kuhn JG Blaney SM Ivy P Sun J Chen Z Adamson PC A phase II trial of rebeccamycin analogue (NSC #655649) in children with solid tumors: a Children's Oncology Group study Pediatric blood & cancer 2008 50 577 580 10.1002/pbc.21274 17610262

[B50] Mita MM Mita AC Chu QS Rowinsky EK Fetterly GJ Goldston M Patnaik A Mathews L Ricart AD Mays T Knowles H Rivera VM Kreisberg J Bedrosian CL Tolcher AW Phase I trial of the novel mammalian target of rapamycin inhibitor deforolimus (AP23573; MK-8669) administered intravenously daily for 5 days every 2 weeks to patients with advanced malignancies Journal of clinical oncology: official journal of the American Society of Clinical Oncology 2008 26 361 367 10.1200/JCO.2007.12.0345 18202410

[B51] Chawla SP Chua VS Fernandez L Quon D Saralou A Blackwelder WC Hall FL Gordon EM Phase I/II and phase II studies of targeted gene delivery in vivo: intravenous Rexin-G for chemotherapy-resistant sarcoma and osteosarcoma Molecular therapy: the journal of the American Society of Gene Therapy 2009 17 1651 1657 19532136 10.1038/mt.2009.126 PMC2835268

[B52] Chugh R Wathen JK Maki RG Benjamin RS Patel SR Meyers PA Priebat DA Reinke DK Thomas DG Keohan ML Samuels BL Baker LH Phase II multicenter trial of imatinib in 10 histologic subtypes of sarcoma using a bayesian hierarchical statistical model Journal of clinical oncology: official journal of the American Society of Clinical Oncology 2009 27 3148 3153 10.1200/JCO.2008.20.5054 19451433

[B53] Widemann BC Goodspeed W Goodwin A Fojo T Balis FM Fox E Phase I trial and pharmacokinetic study of ixabepilone administered daily for 5 days in children and adolescents with refractory solid tumors Journal of clinical oncology: official journal of the American Society of Clinical Oncology 2009 27 550 556 19075272 10.1200/JCO.2008.17.6644 PMC2645861

[B54] Jacobs S Fox E Krailo M Hartley G Navid F Wexler L Blaney SM Goodwin A Goodspeed W Balis FM Adamson PC Widemann BC Phase II trial of ixabepilone administered daily for five days in children and young adults with refractory solid tumors: a report from the children's oncology group Clinical cancer research: an official journal of the American Association for Cancer Research 2010 16 750 754 10.1158/1078-0432.CCR-09-1906 20068084 PMC3086796

[B55] Kurzrock R Patnaik A Aisner J Warren T Leong S Benjamin R Eckhardt SG Eid JE Greig G Habben K McCarthy CD Gore L A phase I study of weekly R1507, a human monoclonal antibody insulin-like growth factor-I receptor antagonist, in patients with advanced solid tumors Clinical cancer research: an official journal of the American Association for Cancer Research 2010 16 2458 2465 10.1158/1078-0432.CCR-09-3220 20371689

[B56] Olmos D Postel-Vinay S Molife LR Okuno SH Schuetze SM Paccagnella ML Batzel GN Yin D Pritchard-Jones K Judson I Worden FP Gualberto A Scurr M de Bono JS Haluska P Safety, pharmacokinetics, and preliminary activity of the anti-IGF-1R antibody figitumumab (CP-751,871) in patients with sarcoma and Ewing's sarcoma: a phase 1 expansion cohort study The lancet oncology 2010 11 129 135 10.1016/S1470-2045(09)70354-7 20036194 PMC2941877

[B57] Kleinerman ES Jia SF Griffin J Seibel NL Benjamin RS Jaffe N Phase II study of liposomal muramyl tripeptide in osteosarcoma: the cytokine cascade and monocyte activation following administration Journal of clinical oncology: official journal of the American Society of Clinical Oncology 1992 10 1310 1316 1634921 10.1200/JCO.1992.10.8.1310

[B58] Kleinerman ES Gano JB Johnston DA Benjamin RS Jaffe N Efficacy of liposomal muramyl tripeptide (CGP 19835A) in the treatment of relapsed osteosarcoma American journal of clinical oncology 1995 18 93 99 10.1097/00000421-199504000-00001 7900714

[B59] Kleinerman ES Meyers PA Raymond AK Gano JB Jia SF Jaffe N Combination therapy with ifosfamide and liposome-encapsulated muramyl tripeptide: tolerability, toxicity, and immune stimulation Journal of immunotherapy with emphasis on tumor immunology: official journal of the Society for Biological Therapy 1995 17 181 193 10.1097/00002371-199504000-00007 7613644

[B60] Harris MB Cantor AB Goorin AM Shochat SJ Ayala AG Ferguson WS Holbrook T Link MP Treatment of osteosarcoma with ifosfamide: comparison of response in pediatric patients with recurrent disease versus patients previously untreated: a Pediatric Oncology Group study Medical and pediatric oncology 1995 24 87 92 10.1002/mpo.2950240205 7990769

[B61] Patel SR Papadopoulos NE Plager C Linke KA Moseley SH Spirindonidis CH Benjamin R Phase II study of paclitaxel in patients with previously treated osteosarcoma and its variants Cancer 1996 78 741 744 10.1002/(SICI)1097-0142(19960815)78:4<741::AID-CNCR8>3.0.CO;2-H 8756366

[B62] Gentet JC Brunat-Mentigny M Demaille MC Pein F Avet-Loiseau H Berger C De Lumley L Pacquement H Schmitt C Sariban E Pillon P Bernard JL Kalifa C Ifosfamide and etoposide in childhood osteosarcoma. A phase II study of the French Society of Paediatric Oncology European journal of cancer 1997 33 232 237 10.1016/S0959-8049(96)00439-X 9135494

[B63] Worth LL Jaffe N Benjamin RS Papadopoulos NE Patel S Raymond AK Jia SF Rodriguez C Gano J Gianan MA Kleinerman ES Phase II study of recombinant interleukin 1alpha and etoposide in patients with relapsed osteosarcoma Clinical cancer research: an official journal of the American Association for Cancer Research 1997 3 1721 1729 9815556

[B64] Voute PA Souhami RL Nooij M Somers R Cortes-Funes H van der Eijken JW Pringle J Hogendoorn PC Kirkpatrick A Uscinska BM van Glabbeke M Machin D Weeden S A phase II study of cisplatin, ifosfamide and doxorubicin in operable primary, axial skeletal and metastatic osteosarcoma. European Osteosarcoma Intergroup (EOI) Annals of oncology: official journal of the European Society for Medical Oncology/ESMO 1999 10 1211 1218 10.1023/A:1008361612767 10586339

[B65] Fagioli F Aglietta M Tienghi A Ferrari S Brach del Prever A Vassallo E Palmero A Biasin E Bacci G Picci P Madon E High-dose chemotherapy in the treatment of relapsed osteosarcoma: an Italian sarcoma group study Journal of clinical oncology: official journal of the American Society of Clinical Oncology 2002 20 2150 2156 10.1200/JCO.2002.08.081 11956277

[B66] Laverdiere C Kolb EA Supko JG Gorlick R Meyers PA Maki RG Wexler L Demetri GD Healey JH Huvos AG Goorin AM Bagatell R Ruiz-Casado A Guzman C Jimeno J Harmon D Phase II study of ecteinascidin 743 in heavily pretreated patients with recurrent osteosarcoma Cancer 2003 98 832 840 10.1002/cncr.11563 12910529

[B67] McTiernan A Whelan JS A Phase II Study of Docetaxel for the Treatment of Recurrent Osteosarcoma Sarcoma 2004 8 71 76 10.1155/2004/762736 18521398 PMC2395610

[B68] Ferrari S Smeland S Mercuri M Bertoni F Longhi A Ruggieri P Alvegard TA Picci P Capanna R Bernini G Muller C Tienghi A Wiebe T Comandone A Bohling T Del Prever AB Brosjo O Bacci G Saeter G Neoadjuvant chemotherapy with high-dose Ifosfamide, high-dose methotrexate, cisplatin, and doxorubicin for patients with localized osteosarcoma of the extremity: a joint study by the Italian and Scandinavian Sarcoma Groups Journal of clinical oncology: official journal of the American Society of Clinical Oncology 2005 23 8845 8852 10.1200/JCO.2004.00.5785 16246977

[B69] Arpaci F Ataergin S Ozet A Erler K Basbozkurt M Ozcan A Komurcu S Ozturk B Celasun B Kilic S Kuzhan O The feasibility of neoadjuvant high-dose chemotherapy and autologous peripheral blood stem cell transplantation in patients with nonmetastatic high grade localized osteosarcoma: results of a phase II study Cancer 2005 104 1058 1065 10.1002/cncr.21279 15999369

[B70] McTiernan A Meyer T Michelagnoli MP Lewis I Whelan JS A phase I/II study of doxorubicin, ifosfamide, etoposide and interval methotrexate in patients with poor prognosis osteosarcoma Pediatric blood & cancer 2006 46 345 350 10.1002/pbc.20562 16206197

[B71] Seibel NL Krailo M Chen Z Healey J Breitfeld PP Drachtman R Greffe B Nachman J Nadel H Sato JK Meyers PA Reaman GH Upfront window trial of topotecan in previously untreated children and adolescents with poor prognosis metastatic osteosarcoma: children's Cancer Group (CCG) 7943 Cancer 2007 109 1646 1653 10.1002/cncr.22553 17334983

[B72] Basaran M Bavbek ES Saglam S Eralp L Sakar B Atalar AC Bilgic B Ozger H Onat H A phase II study of cisplatin, ifosfamide and epirubicin combination chemotherapy in adults with nonmetastatic and extremity osteosarcomas Oncology 2007 72 255 260 10.1159/000113017 18185020

[B73] Iwamoto Y Tanaka K Isu K Kawai A Tatezaki S Ishii T Kushida K Beppu Y Usui M Tateishi A Furuse K Minamizaki T Kawaguchi N Yamawaki S Multiinstitutional phase II study of neoadjuvant chemotherapy for osteosarcoma (NECO study) in Japan: NECO-93J and NECO-95J Journal of orthopaedic science: official journal of the Japanese Orthopaedic Association 2009 14 397 404 19662473 10.1007/s00776-009-1347-6

[B74] Berger M Grignani G Ferrari S Biasin E Brach del Prever A Aliberti S Saglio F Aglietta M Fagioli F Phase 2 trial of two courses of cyclophosphamide and etoposide for relapsed high-risk osteosarcoma patients Cancer 2009 115 2980 2987 10.1002/cncr.24368 19452540

[B75] Kolb EA Kushner BH Gorlick R Laverdiere C Healey JH LaQuaglia MP Huvos AG Qin J Vu HT Wexler L Wolden S Meyers PA Long-term event-free survival after intensive chemotherapy for Ewing's family of tumors in children and young adults Journal of clinical oncology: official journal of the American Society of Clinical Oncology 2003 21 3423 3430 10.1200/JCO.2003.10.033 12972518

[B76] Meyer T McTiernan A Whelan J A Phase II Study of Docetaxel in Patients with Relapsed and Refractory Ewing's Tumours Sarcoma 2003 7 13 17 10.1080/1357714031000114192 18521364 PMC2395514

[B77] Bernstein ML Devidas M Lafreniere D Souid AK Meyers PA Gebhardt M Stine K Nicholas R Perlman EJ Dubowy R Wainer IW Dickman PS Link MP Goorin A Grier HE Intensive therapy with growth factor support for patients with Ewing tumor metastatic at diagnosis: Pediatric Oncology Group/Children's Cancer Group Phase II Study 9457--a report from the Children's Oncology Group Journal of clinical oncology: official journal of the American Society of Clinical Oncology 2006 24 152 159 10.1200/JCO.2005.02.1717 16382125

[B78] Rosenthal J Bolotin E Shakhnovits M Pawlowska A Falk P Qian D Oliver C Sato J Miser J Forman S High-dose therapy with hematopoietic stem cell rescue in patients with poor prognosis Ewing family tumors Bone marrow transplantation 2008 42 311 318 10.1038/bmt.2008.169 18587438

[B79] DuBois SG Krailo MD Lessnick SL Smith R Chen Z Marina N Grier HE Stegmaier K Phase II study of intermediate-dose cytarabine in patients with relapsed or refractory Ewing sarcoma: a report from the Children's Oncology Group Pediatric blood & cancer 2009 52 324 327 10.1002/pbc.21822 18989890 PMC2791370

[B80] Casey DA Wexler LH Merchant MS Chou AJ Merola PR Price AP Meyers PA Irinotecan and temozolomide for Ewing sarcoma: the Memorial Sloan-Kettering experience Pediatric blood & cancer 2009 53 1029 1034 10.1002/pbc.22206 19637327

